# Supplementary material for: Field-free spin-orbit torque-induced switching of perpendicular magnetization in a ferrimagnetic layer with a vertical composition gradient
Source: Nat Commun. 2021 Jul 27;12:4555. doi: 10.1038/s41467-021-24854-7 (PMC8316453; doi:10.1038/s41467-021-24854-7)
Supplement: Supplementary file 1 — Supplementary Information [file 41467_2021_24854_MOESM1_ESM.pdf]

Supplementary Information

Field-free spin-orbit torque-induced switching of perpendicular magnetization in a ferrimagnetic layer with vertical composition gradient

Zhenyi Zheng[1,2,3,7], Yue Zhang[1,7,*], Victor Lopez-Dominguez[2], Luis Sánchez-Tejerina[4], Jiacheng Shi[2], Xueqiang Feng[1], Lei Chen[1], Zilu Wang[1], Zhizhong Zhang[1], Kun Zhang[1], Bin Hong[1], Yong Xu[1], Youguang Zhang[3], Mario Carpentieri[5], Albert Fert[1,6], Giovanni Finocchio[4,*], Weisheng Zhao[1,*], Pedram Khalili Amiri[2,*]

[1]Fert Beijing Research Institute, School of Integrated Circuit Science and Engineering, Beijing Advanced Innovation Center for Big Data and Brain Computing, Beihang University, Beijing, P. R. China

[2]Department of Electrical and Computer Engineering, Northwestern University, Evanston, IL, USA

[3]School of Electronics and Information Engineering, Beihang University, Beijing, P. R. China

[4]Department of Mathematical and Computer Sciences, Physical Sciences and Earth Sciences, University of Messina, Messina, Italy

[5]Dipartimento di Ingegneria Elettrica e dell'Informazione, Politecnico di Bari, Bari, Italy

[6]Unité Mixte de Physique, CNRS, Thales, Univ. Paris-Sud, University of Paris-Saclay, Palaiseau, France

[7]These authors contributed equally: Zhenyi Zheng, Yue Zhang.

[*]e-mail: yz@buaa.edu.cn, gfinocchio@unime.it, weisheng.zhao@buaa.edu.cn, pedram@northwestern.edu.

S1. Current density distribution inside the material stack

Here we show the method used to estimate the current density distribution in the designed device. The resistance of every sample with different Tb composition gradient δ was firstly measured. As plotted in Fig. S1a, with δ rising from 0.00 to 0.07, the device resistance R increases from 223 Ω to 318 Ω. This 43% increase indicates that the resistivity of CoTb should have a large change, since the Al layer is unchanged. We then directly measured the resistivity of $Co_{0.87}Tb_{0.13}$ and $Co_{0.52}Tb_{0.48}$ films, which were determined to be 104 μΩ-cm and 385 μΩ-cm, respectively.

Assuming that the resistivity of CoTb has a linear relationship between these two compositions, the resistivity of each composition in our experiments can then be calculated. Considering the parallel resistance model and the device dimensions, the theoretical resistance value of each device with varying δ was determined and is plotted in Fig. S1a. A good agreement is observed between the experimental values and those from our model, where the small resistance shift can be attributed to the gold electrode resistance and contact resistances. For each sample with different gradient, the current density distributions in the CoTb layer and the Al layers were calculated by the standard parallel resistance model. We apply this method in both harmonics measurements and critical switching current density estimations.

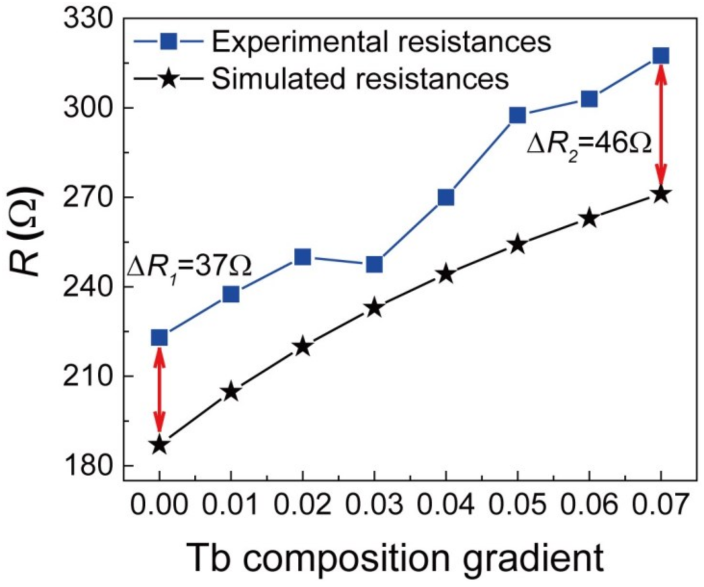

Fig. S1 Device resistance. A comparison between the experimental and calculated resistances of the device as a function of composition gradient δ.

S2. Dependence of critical current density on the applied magnetic field

Here we show the magnetic-field dependent critical switching current density j_c in the sample with $\delta = \pm 0.07$. In the theoretical SOT framework, j_c could be expressed as [1]

$$j_c = \frac{2e}{\hbar}\frac{M_S t_M}{\theta_{SH}}\left(\frac{H_{K,eff}}{2} - \frac{H_x}{\sqrt{2}}\right), \quad \text{(S1)}$$

where M_s, t_M, $H_{K,eff}$, H_x, θ_{SH} are the net magnetization, thickness of the magnetic layer, effective magnetic anisotropy field, applied in-plane field and effective spin Hall angle, respectively. From Equation S1, j_c is predicted to decrease if a larger H_x is applied. Fig. S1 summarizes j_c as a function of H_x for the two samples with $\delta = \pm 0.07$, respectively. Clearly, field-free switching demands the largest j_c in all cases.

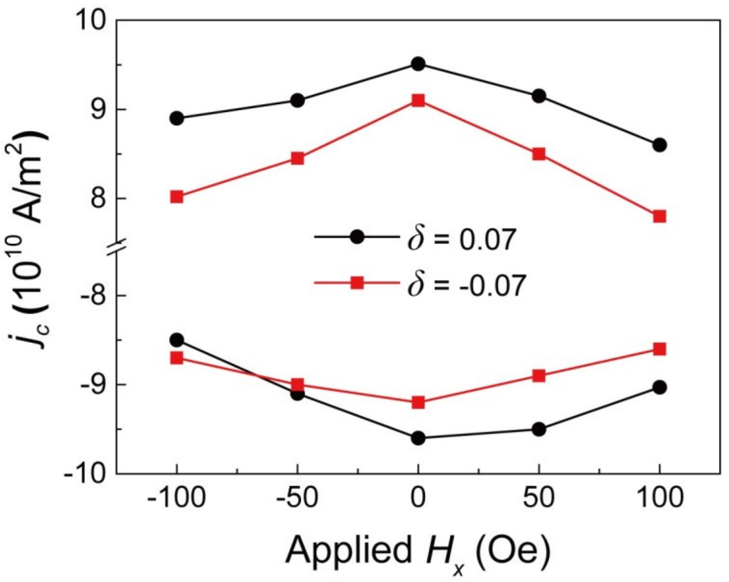

Fig. S2 Critical SOT switching current density as a function of in-plane magnetic field. j_c arrives at the peak value when the magnetic field is absent for samples with $\delta = \pm 0.07$.

S3. SOT switching and DMI for the 6 nm sample with δ = 0.06

In addition to $\delta = \pm 0.07$, we also carried out SOT switching experiments in a sample with δ = 0.06 and 6 nm overall CoTb thickness, to demonstrate the generality of the DMI-induced field-free switching phenomenon. Fig. S3a illustrates the switching loops under different magnetic fields from 40 Oe to -40 Oe along the current direction. A clear magnetization switching in the absence of the in-plane magnetic field is observed, which has the same polarity as that under positive magnetic field. This property is similar to what we saw from the sample with δ = 0.07 in the main text. Besides, the deterministic switching vanishes at -10 Oe, which corresponds well to the DMI effective field (~ 12 Oe) measured in Fig. S3b by the method mentioned in the main text. This verifies again that g-DMI dominates the switching polarity in the absence of any in-plane field.

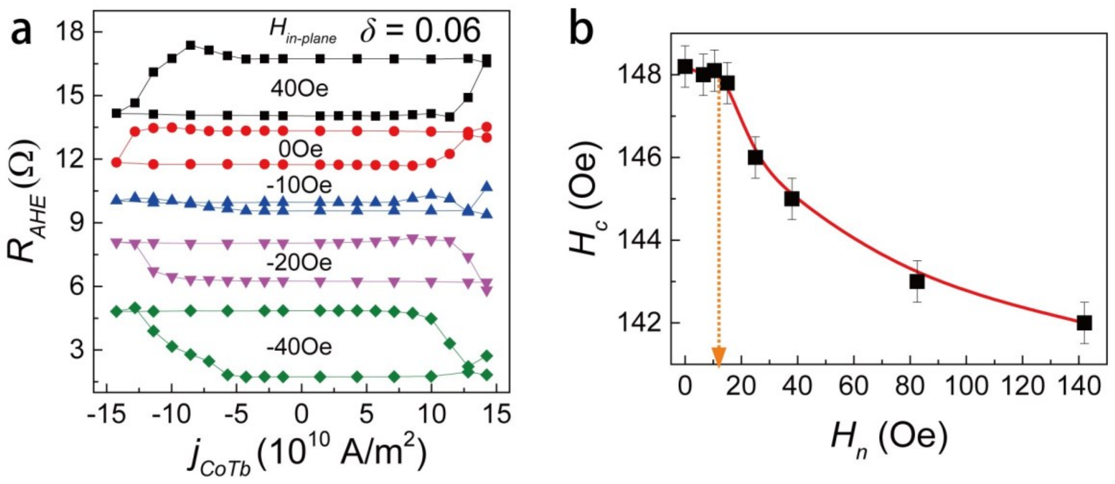

Fig. S3 SOT switching and DMI measurement for the device with δ = 0.06. a, AHE resistance versus applied current density j_{CoTb} under varying in-plane magnetic fields **b,** DMI effective field measurement. The threshold value of H_n, which is at the end of the platform, can be treated as the DMI effective field. Error bars are obtained from repeated measurements for each angle of applied magnetic field.

S4. SOT switching in a CoTb sample with 9 nm thickness

We primarily focus on samples with a fixed thickness, i.e. 6 nm, in the main text. Here, we show that effective field-free switching can also be achieved in samples with a larger thickness. Fig. S4a shows the structure for a 9 nm thick CoTb sample. The thickness of each CoTb layer with different composition was increased from 1 nm to 1.5 nm compared to earlier experiments, while the composition step change (0.07) between two adjacent layers remained the same. Fig. S4b shows the switching loop of the described sample under different in-plane fields. We can see that field-free switching also occurs in this sample.

In addition, one interesting point is the critical switching current density j_c change. The j_c of this 9 nm thick sample and of the sample in Supplementary Note S3 are around 12×10^{10}A/m^2 and 11×10^{10}A/m^2, respectively. Both values are larger than the j_c in the 6 nm thick CoTb sample with δ = 0.07 (~ 9×10^{10}A/m^2). This result is in good agreement with our observed spin Hall angle trend in the main text, since the absolute composition gradients per nanometer for both of these samples are smaller than that in the 6 nm thick CoTb sample with δ = 0.07.

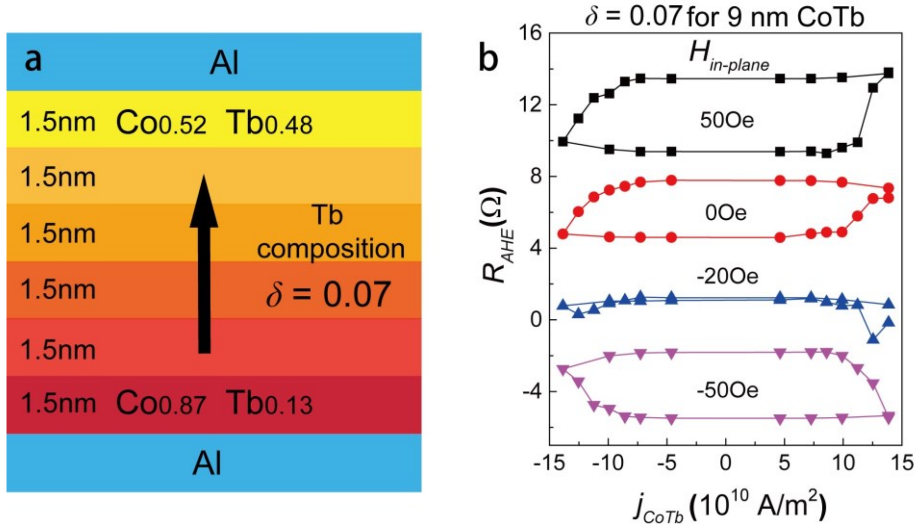

Fig. S4 Stack structure and SOT switching of the 9 nm thick CoTb device. a, Stack structure of the 9-nm-thick film. The only difference of this film and the 6-nm-thick CoTb sample with δ = 0.07 is that the thickness of the six CoTb layers increases by 50%. **b,** SOT switching curves of this sample under different in-plane magnetic fields.

S5. SOT switching in a CoTb sample with 4.2 nm thickness

Here we demonstrate that a similar field-free switching can also be achieved in a thinner CoTb film with a steeper composition gradient. In this case, we reduce the thickness of each CoTb layer to 0.7 nm, while keeping the composition step change between adjacent layers at $\delta = 0.07$. As a result, the total CoTb thickness (consisting of six layers) is 4.2 nm, while the effective slope of the composition gradient is larger than in all the other samples.

Fig. S5a shows the switching loops as a function of j_{CoTb} for this sample. Field-free switching is achieved, and the critical switching current density is reduced to ~7.5×10^{10}A/m^2. In addition, the switching disappears at an external field of around 40 Oe, which corresponds well to the value of H_{DMI} determined in Fig. S5b. The relatively smaller critical switching current and the larger H_{DMI} can be explained by the larger absolute composition gradient in this sample.

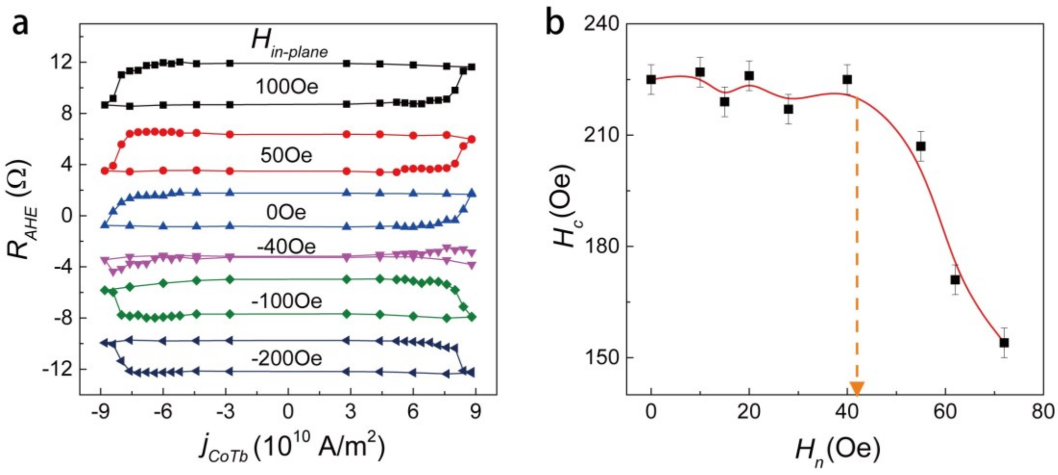

Fig. S5 SOT switching and DMI measurement for a device with 4.2 nm total CoTb thickness. **a,** AHE resistance versus applied current density j_{CoTb} under varying in-plane magnetic fields. **b,** DMI effective field measurement. The DMI effective field of this sample is approximately 40 Oe. Error bars are obtained from repeated measurements for each angle of applied magnetic field.

S6. Micromagnetic framework

The micromagnetic solver is based on the solution of two Landau-Lifshitz-Gilbert (LLG) equations strongly coupled by exchange interactions [2-6]

$$\begin{cases} \frac{d\boldsymbol{m}_1}{dt} = -\gamma_1 \mathbf{m}_1 \times \mathbf{H}_{eff,1} + \alpha \mathbf{m}_1 \times \frac{d\mathbf{m}_1}{dt} + \boldsymbol{\tau}_{SH,1} \\ \frac{d\boldsymbol{m}_2}{dt} = -\gamma_2 \mathbf{m}_2 \times \mathbf{H}_{eff,2} + \alpha \mathbf{m}_2 \times \frac{d\mathbf{m}_2}{dt} + \boldsymbol{\tau}_{SH,2} \end{cases} \quad \text{(S2)}$$

where $\mathbf{m}_i$ is the sublattice magnetization vector, γ_i is the gyromagnetic ratio of the i-th sublattice, and α is the Gilbert damping parameter (same for both sub-lattices). The intrinsic SOT is taken into account as an additional damping-like torque term [7]

$$\boldsymbol{\tau}_{SH,i} = -\gamma_i H_{SH,i} \mathbf{m}_i \times (\mathbf{m}_i \times \mathbf{p}) \quad \text{(S3)}$$

whose amplitude is given by $H_{SH,i} = \hbar J \theta_{SH} / 2et\mu_0 M_{S,i}$. In the last expression, $\hbar$, θ_{SH}, e, t, μ_0 are the reduced Planck's constant, the intrinsic spin Hall angle, the electron charge, the film thickness, and the vacuum permeability respectively, while J is the applied current density. $M_{S,i}$ is the saturation magnetization of the i-th sublattice. The direction of the spin Hall polarization is $\mathbf{p} = \mathbf{z} \times \mathbf{j}$, $\mathbf{j}$ being the unit vector of the current density direction which is assumed to be along the x direction (see Fig. 2a in the main text). $H_{SH,1}$ and $H_{SH,2}$ are the effective fields for the first and second sublattice, respectively. The main contributions to these fields are the exchange interactions, the perpendicular uniaxial magnetic anisotropy and the g-DMI. The contributions from the first two fields are given by

$$\begin{cases} \mathbf{H}_{exch,i} = \frac{2A_{11}}{\mu_0 M_{S,i}} \nabla^2 \mathbf{m}_i + \frac{4A_0}{a^2 \mu_0 M_{S,i}} \mathbf{m}_j + \frac{A_{12}}{\mu_0 M_{S,i}} \nabla^2 \mathbf{m}_j \\ \mathbf{H}_{anis,i} = \frac{2K_u}{\mu_0 M_{S,i}} m_{z,j} \mathbf{u}_z \end{cases}, \quad \text{(S4)}$$

Where i ≠ j, a is the magnetic lattice constant and A_{11}, A_0, A_{12} are the inhomogeneous intra-lattice, homogeneous inter-lattice and inhomogeneous inter-lattice exchange constants characterizing the interaction, and finally $K_{u,i}$ is the perpendicular anisotropy constant. Additionally, although the DMI appears on the bulk of the CoTb, it originates from gradient-driven symmetry breaking along the **z** direction, similar to interfacial DMI. Therefore, the g-DMI field has the same expression as the interfacial DMI [8-10]

$$\mathbf{H}_{DMI,i} = \frac{-2D}{\mu_0 M_{S,i}}(\mathbf{u}_z \cdot (\nabla \cdot \mathbf{m}_i) - \nabla m_{z,i}), \tag{S5}$$

with the following boundary conditions [5,10]

$$2A_{11}\partial_n \mathbf{m}_i + A_{12}\mathbf{m}_i \times (\partial_n \mathbf{m}_j \times \mathbf{m}_i) + D\mathbf{m}_i \times (\mathbf{n} \times \mathbf{u}_z) = \mathbf{0}. \tag{S6}$$

We have chosen for the first (second) sublattice the Co (Tb) lattice in terms of physical parameters. However, the simulation results are the same when reversing the choice. We have discretized the device geometry with tetragonal cells of 10×10×6 nm^3, considering the parameters averaged along the *z*-direction. The device geometry is shown in Fig. S6. The wider arm is of 200 nm width and 600 nm length and the narrower arm has a 100 nm width. The device thickness is 6 nm.

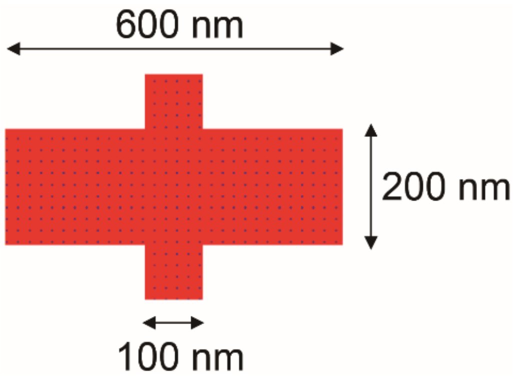

Fig. S6 Geometrical description of the simulated device. The device was discretized with tetragonal cells of 10×10×6 nm^3.

The effective field was augmented with an additional Gaussian random field $H_{th,i}$, to mimic the effect of the finite temperature

$$\mathbf{H}_{th,i} = \boldsymbol{\eta}\sqrt{\frac{2\alpha(1+\alpha^2 k_B T)}{\gamma_0 \mu_0 M_s V dt}}, \tag{S7}$$

With k_B, T and V being the Boltzmann constant, the temperature (set to 300 K in our case) and the computational cell volume, respectively. In this equation, $\boldsymbol{\eta}$ is a vector whose Cartesian components are random numbers following the Gaussian distribution:

$$\begin{cases} \langle \eta_k(t) \rangle = 0 \\ \langle \eta_k(t)\eta_l(t) \rangle = \delta_{kl}\delta(\mathbf{r}-\mathbf{r}')\delta(t-t') \end{cases}. \tag{S8}$$

Finally, most of the simulation parameters were derived from the experiments in this work or from other experimental works on similar material systems, except for the exchange and lattice constants. Table S1 summarizes the detailed parameters.

Table S1 Summary of simulation parameters.

Parameter	Value	Source
M_{S1}	654 kA/m	This work (see Fig. 1d). We assumed Co magnetization to be independent of Tb composition.
M_{S2}	609 kA/m	This work (see Fig. 1d). We assumed Tb magnetization to be independent of Co composition.
K_{u1}; K_{u2}	20 kJ/m^3	This work, assuming $K_{u1} = K_{u2}$. Note that the effective value is the sum.
D	16 μJ/m^2	This work (see Fig. 3).
A_{11}	3 pJ/m	Assumed
A_{12}	-3 pJ/m	Assumed
A_0	-6 pJ/m	Assumed
θ_{SH}	0.06	This work (see Fig. 2d).
γ_1	2.42×10^5 m/As	Literature [11-12].
γ_2	1.69×10^5 m/As	Literature [11-12].
α	0.05	Assumed

S7. Additional micromagnetic simulations

Fig. S7 a-h depict a sublattice magnetization under the effect of a negative current pulse (the same timing as in Fig. 5). It can be clearly observed that the switching dynamics are similar to (but mirrored) the positive current pulses.

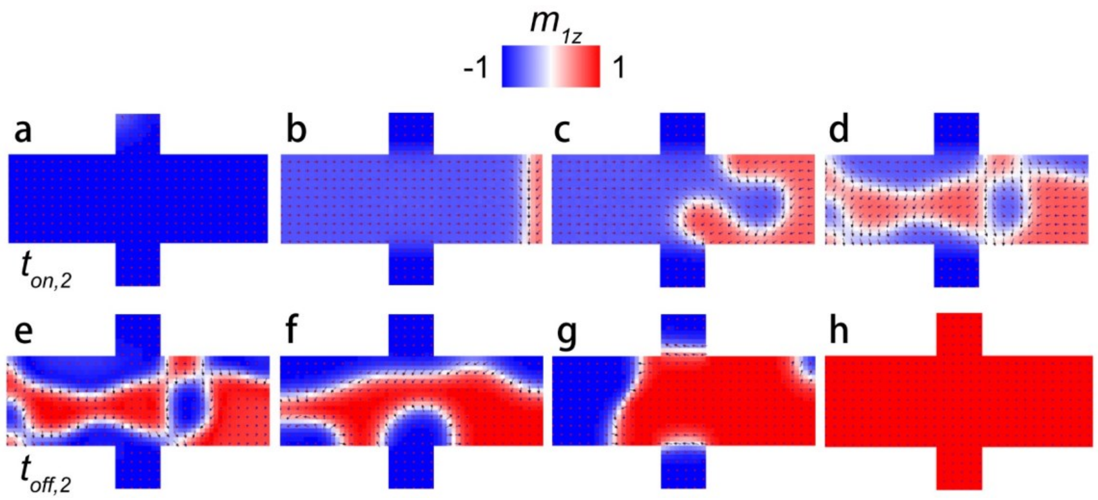

Fig. S7 Micromagnetic simulations in a Hall bar. a-h, First sublattice magnetization distribution at different times of the dynamics (from 32 ns to 64 ns).

To study the scalability to a realistic memory device geometry, we also performed similar simulations for a pillar with a diameter of 400 nm. Fig. S8 a-j depict the sublattice magnetization under a positive current pulse (being applied from 0 ns to 20 ns). The switching dynamics are qualitatively similar to those observed in the case of the Hall bars.

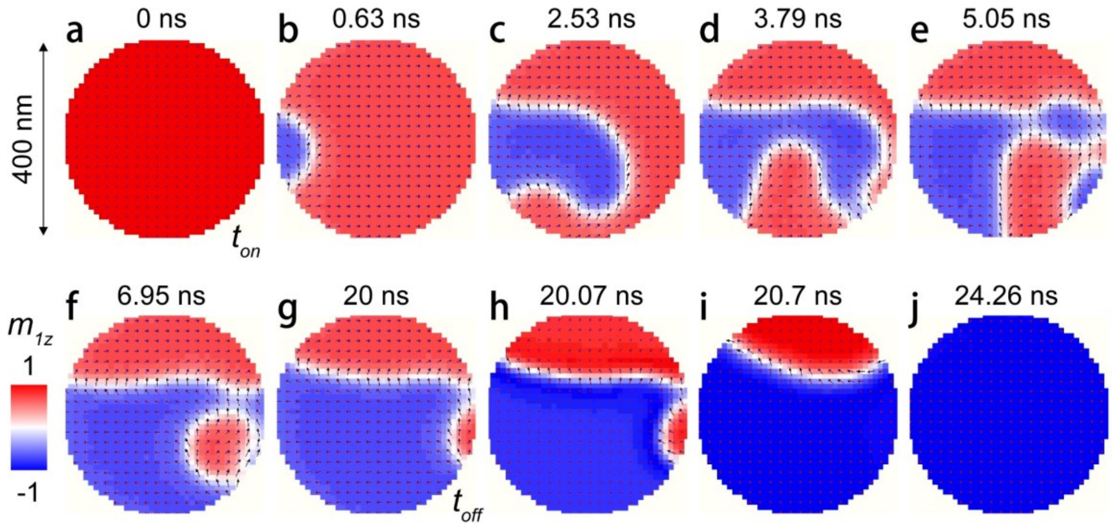

Fig. S8 Micromagnetic simulations in a pillar with 400 nm diameter. Panels a-j show the first sublattice magnetization distribution at different times of the switching dynamics in the pillar.

S8. Comparison of the switching efficiency

This Supplementary Note compares the field-free deterministic SOT switching efficiency in our CoTb samples with: (i) previous reports of SOT-induced switching in ferrimagnetic (FIM) CoTb films (where a bias magnetic field was used to achieve deterministic switching) [12-15]; and (ii) previous reports of field-free deterministic SOT switching of perpendicular ferromagnetic (FM) layers, specifically CoFeB, Co, and Co/Ni/Co [16-19].

Two figures of merit are used for the comparison: The first is the switching current density itself (j_c), which in the case of our 6 nm CoTb samples, is ~ 9 MA/cm^2 as discussed in the main text. The second figure of merit is the switching current density divided by the energy barrier between the two magnetic states, normalized by surface area, i.e. $j_c / (t \cdot K_{eff})$. Here t is the (ferromagnetic or ferrimagnetic) film thickness, and K_{eff} is its effective perpendicular anisotropy energy density. The latter figure of merit, in principle, provides a fairer basis for comparison, since the difference in switching current densities of different material systems is normalized by their energy barrier, which would determine the data retention time in a magnetic memory element [20-22].

The result of this comparison is shown in Table S2. It is evident that the vertical gradient CoTb structure has the best figure of merit $j_c / (t \cdot K_{eff})$, by ~ 3× to 6×, as well as comparable or better (up to ~9× smaller) switching current density, when compared to previous reports of field-free SOT switching in ferromagnetic material structures with in-plane static symmetry-breaking. Our gradient CoTb structure also performs best on both figures of merit, when compared to previous reports of SOT switching in CoTb (all of which required an in-plane bias magnetic field, unlike the structures presented in this work). In the comparison with reference [15] where a single CoTb layer is used without adjacent heavy metals, we only compared j_c because of the lack of K_{eff} data. These comparisons directly demonstrate the high switching efficiency brought about by the engineered composition gradient.

The superior SOT switching efficiency in the CoTb samples with a vertical gradient can be qualitatively understood as follows. Given that the combination of

SOT and g-DMI that results in the deterministic switching is created by the gradient-induced broken inversion symmetry along the film thickness, it is present throughout the volume of the material, even in relatively thick films (4.2 nm to 9 nm in our case). As a result, our switching current density is smaller than in previous interfacial SOT switching experiments in the CoTb material system, and can further be tailored by engineering the slope of the concentration gradient within the film, which determines its spin Hall angle. This, combined with the increased film thickness over which deterministic switching can be achieved, result in the significant improvement of the SOT efficiency figure of merit $j_c / (t \cdot K_{eff})$.

Table S2 Comparison of SOT switching efficiency. The CoTb vertical gradient structure presented in this work provides the best SOT efficiency figure of merit, when compared to both: (i) previous reports of SOT switching in CoTb films (with a bias magnetic field, H_{ex}); and (ii) previous reports of field-free deterministic SOT switching of perpendicular ferromagnetic layers.

**Structure**	t [nm]	K_{eff} [J/m^3]	j_c [MA/cm^2]	$j_c / (t \cdot K_{eff})$ [10^{14} A/J]	H_{ex} [Oe]	**FL Type**
CoTb (vertical gradient) [this work]	6	4.9×10^4	9	3.1	0	FIM
Pt/CoTb [13]	5	7.5×10^4	40	10.7	200	FIM
Ta/CoTb [12]	2	6.4×10^4	15	15.6	2000	FIM
W/CoTb [14]	3.5	2×10^5	45	6.4	100	FIM
$SiN_x/Co_{0.61}Tb_{0.39}/SiN_x$ [15]	6	--	13	--	20	FIM
Ta/CoFeB/TaO_x (in-plane gradient) [16]	1	5.0×10^4	5	10	0	FM
Mo (in-plane gradient)/CoFeB/MgO [17]	1.4	2.1×10^5	42	14.3	0	FM
Pt/Co/IrMn (in-plane exchange bias) [18]	0.7	6×10^5	80	19.1	0	FM
PMN-PT (in-plane strain)/Pt/Co/Ni/Co [19]	1	8×10^4	12.5	15.6	0	FM

S9. Role of device orientation and applied current history (current-induced training) in the deterministic field-free switching

We performed additional experiments to investigate the nature of the observed deterministic switching, and correspondingly, to determine the symmetry requirements that would need to be met, to be consistent with the experimental data. These new results and the associated discussion of the experiment's symmetry are given below.

As the first step, we sought to verify that the field-free SOT-induced switching in our devices is indeed directional, i.e. that each current direction favors a particular final state (up or down), and that this directionality is consistent across a large number of devices. To do so, we first performed a time-domain switching measurement. The purpose of this experiment is to illustrate that the final state of the device after switching is determined by the direction of the writing current, but not by the number of consecutive current pulses sent in the same direction, i.e. that the device is not undergoing toggle switching. The results can be seen in Fig. S9, where black pulses indicate write attempts in different directions (0.1 ms, +11 MA/cm^2 or -11 MA/cm^2) and red circles correspond to the subsequent readout, i.e. the measurement of the R_{AHE} of CoTb. Note that we perform two consecutive read attempts in each case, to also verify that the readout process itself, i.e. the measurement of R_{AHE}, does not change the state of the device in any way.

It is clear from Fig. S9 that the first writing pulse in each direction switches the magnetization of the CoTb in the corresponding up/down direction, but that subsequent pulses in the same direction (regardless of their number) do nothing to change the state of the bit. This clearly shows that the applied current pulse polarity (but not the number of pulses in the same direction) changes the R_{AHE} value, indicating that the switching is deterministic and directional. Note that, this behavior is qualitatively different from what Refs. [23-25] (Refs. [33-35] in the main text) predicted from micromagnetic simulations. In those simulations, the writing current, no matter what direction it had, always flipped the bit to the opposite magnetic state,

thus exhibiting toggle switching.

Next, we performed additional experiments to verify whether any (unintentional) in-plane asymmetry may play a role in our observations. To do so, a direct evidence would be whether the in-plane device orientation impacts the switching polarity. To answer this question, we fabricated new samples where devices with different angles are arrayed on the same chip (see Fig. S10). As illustrated in Fig. S11 (left panel), we first measured the SOT switching loops in four different devices with 0 degree, 45 degree, 90 degree and 135 degree configurations, respectively. It is clearly seen that all of them exhibit the same switching polarity. Next, we rotated the measurement setup (without rotating the chip) by 180 degrees in these four devices to measure the switching loops again, corresponding to the 180 degree, 225 degree, 270 degree and 315 degree configurations, as indicated in the right panel of Fig. S11. As would be expected from a directional switching behavior, the switching loops are all inversed in this case. To verify that this inversed switching polarity is repeatable in a single device, we randomly chose a 45 degree device on the chip and measured it with both configurations, i.e. the 45 degree and 225 degree setups shown in Fig. S11, for several times. As shown in Fig. S12, the directionality of the switching can be clearly repeated, and therefore cannot be attributed to any random event.

It cannot be emphasized enough, that the results of Fig. S11 seem to defy the symmetry of the structure. However, our next experiment showed that this is not the case, and in fact all symmetry considerations are indeed satisfied when the experiment is considered in its entirety, including the training effect from the initial current pulse applied to the device.

The only possible symmetry-breaking element between the first four measurements (left panel in Fig. S11) and the second four measurements (right panel in Fig. S11) is that in the second four measurements, current pulses have already been injected in the device before, i.e., the devices have been trained by the first current pulse which has been applied in the positive direction of the device, as defined on the left panel of Fig. S11.

To control for this training variable, we chose several new devices, in which no

current had been applied before subsequent to device fabrication. Fig. S13 shows the corresponding SOT switching loops for three of these devices. Note that the definitions of measurement setup angles in Fig. S13 and Fig. S11 are the same. We notice that in sharp contrast to Fig. S11, in this case all the devices (0 degree, 180 degree and 225 degree) show the same SOT-induced switching directionality. Notably, the cases of 180 degree and 225 degree devices show the opposite directionality compared to Fig. S11. This can be explained based on the training effect provided by the initial current sent through the devices, which is opposite for these two angles in Figs. S11 and S13, and therefore breaks the symmetry of the experiment.

In what follows, we discuss about the possible explanation of these results. Given the size of our devices, it is expected that SOT-induced magnetization switching is completed by domain wall (DW) motion, as is the case in most other SOT-induced switching experiments reported to date. Inspired by this DW-mediated switching character, recently a field-free deterministic switching scheme has been proposed that relies on geometrical pinning of DWs [26]. Similar to our work, the device structure proposed in Ref. [26] also does not break the lateral in-plane structural symmetry; however, its deterministic switching is allowed because of the broken inversion symmetry of the micromagnetic structure of the device.

We hypothesize that in our devices, the first current pulse applied to the device creates a chiral magnetic texture (CMT) due to the simultaneous effect of SOT and g-DMI. For example, this could be in the form of the nucleation of initial DWs with a chirality that depends on the g-DMI, at the edges of the device. These DWs are possibly pinned by defects and maintain their existence during magnetization switching. Similar to Ref. [26], their presence can break the micromagnetic in-plane symmetry of the experiment and in principle allows for deterministic switching to occur.

We performed further experiments, as described below, which provide additional evidence for the creation of such a chiral magnetic texture in response to training by a current pulse. Specifically, we hypothesize that if indeed the presence of a CMT is responsible for the observed training effect, it should be possible to destroy the effect

of this training using a sufficiently large magnetic field applied to the device. This, in turn, would allow one to subsequently retrain the device by applying a new training current pulse in the opposite direction. In addition, if this hypothesis is correct, there should be a range of smaller external magnetic fields which would not be sufficient to destroy the CMT, and hence the training effect. Our experiments clearly confirmed this hypothesis, as described below:

a) We chose a new device where no current pulses had been applied before. This device was first trained with a current pulse (0.1 ms, ~11 MA/cm^2) along the +x direction (as defined in the coordinate axes shown in Fig. S14b).
b) We then performed current-induced switching experiments in this device, using the 0 degree measurement configuration (Fig. S14b, top panel) and the 180 degree measurement configuration (Fig. S14b, bottom panel). Consistent with our previous results, the initial current pulse resulted in training of the device, giving rise to field-free deterministic and directional switching, with the 0 degree and 180 degree measurement configurations resulting in opposite polarities of the loops, as expected.
c) We then applied an out-of-plane magnetic field H_1 to destroy any magnetic texture in the device, thus resetting the device to the initial pre-training state. This was followed by applying a current pulse (0.1 ms, ~11 MA/cm^2) along the –x direction (as defined in the coordinate axes shown in Fig. S14b), to retrain the same device.
d) Subsequent to this retraining attempt, we again performed current-induced switching measurements in the device using both the 0 degree measurement configuration (Fig. S14b, top panel) and the 180 degree measurement configuration (Fig. S14b, bottom panel).

The results of this reset and retraining experiment were as follows:

1. Large H_1 field: Fig. S14a shows the results for the case where the applied *reset* magnetic field H_1 is 8 T. Clearly, after going through an 8 T field and being retrained by a current along the –x direction, the device exhibits the opposite switching directionality compared to the original training.
2. Small H_1 field: Next, we repeated the same sequence of experimental steps in

another (also previously untrained) device, with the only difference being that the applied reset field H_1 was chosen to be smaller (0.2 T). Fig. S14c shows the results for this case, which clearly indicate that the retraining process does not work with this smaller field. In other words, the device keeps its original training and switching directionality after going through a 0.2 T field.

We interpret these results as a strong confirmation that a CMT is responsible for the observed training effect and directional switching in our devices. This CMT can be destroyed by a large field of 8 T, but not by a smaller field of 0.2 T.

It is worth noting that in principle, even without applying an external 8 T field, a large enough current pulse in the opposite direction (compared to the initial training current) should be able to directly modify the CMT and retrain the device to exhibit an opposite switching directionality. In our experiments, however, we were not able to apply sufficiently large currents to do so without burning the device.

Finally, it is worth noting that the concept of a directional anisotropy, defined by the history of applied fields or torques to the magnetization, has previously been observed in other material systems. Specifically, the "nonvolatile chirality printing" seen here is in some ways similar to the so-called "triad anisotropy", previously studied in disordered magnetic alloys (spin glasses) exhibiting DMI induced by heavy impurities with large spin-orbit coupling [27-31]. In those cases, however, the emergence of a directional "triad anisotropy" was the result of the history of applied magnetic fields in conjunction with DMI, unlike our present case where the training occurs in response to SOT and DMI.

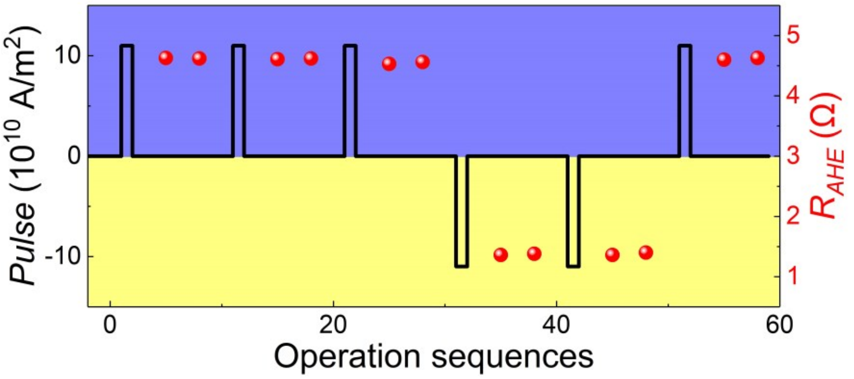

Fig. S9 Time-domain measurement with writing pulses in both directions, demonstrating that the switching is deterministic and directional.

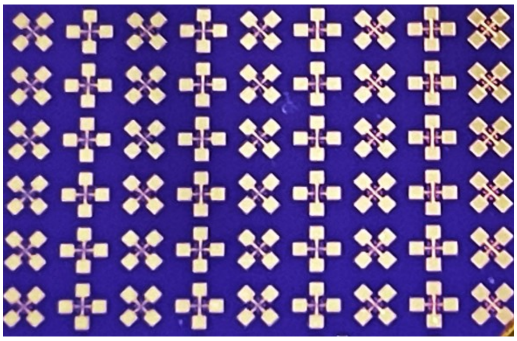

Fig. S10 Optical image of the fabricated sample where devices with different angles are arrayed.

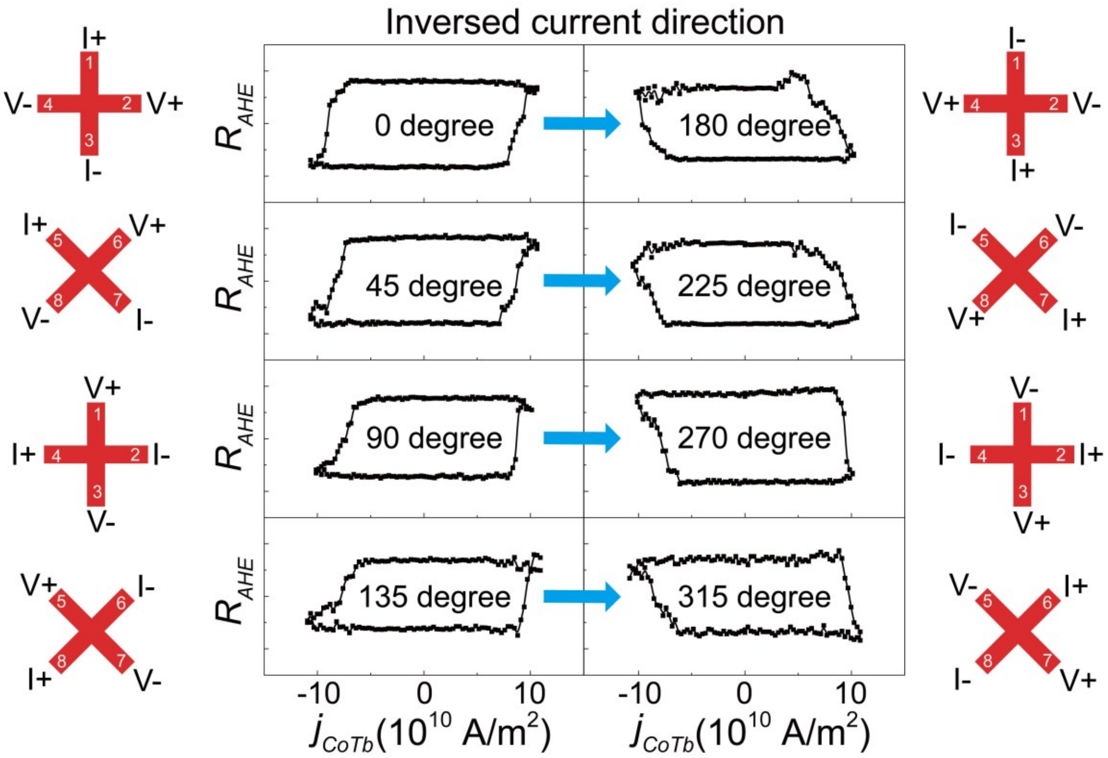

Fig. S11 SOT switching loops of devices varying from 0 degree to 135 degree and the corresponding loop of each device with a 180 degrees inversed measurement setup.

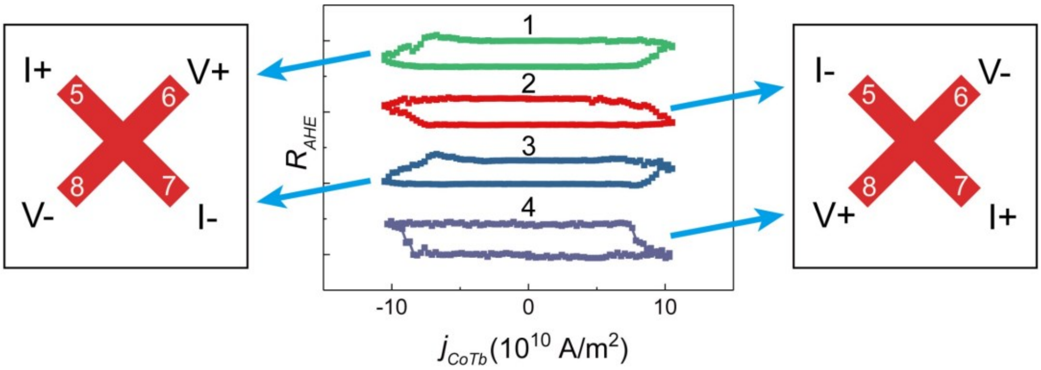

Fig. S12 SOT switching experiment repetitions with two 180 degree rotated setups.

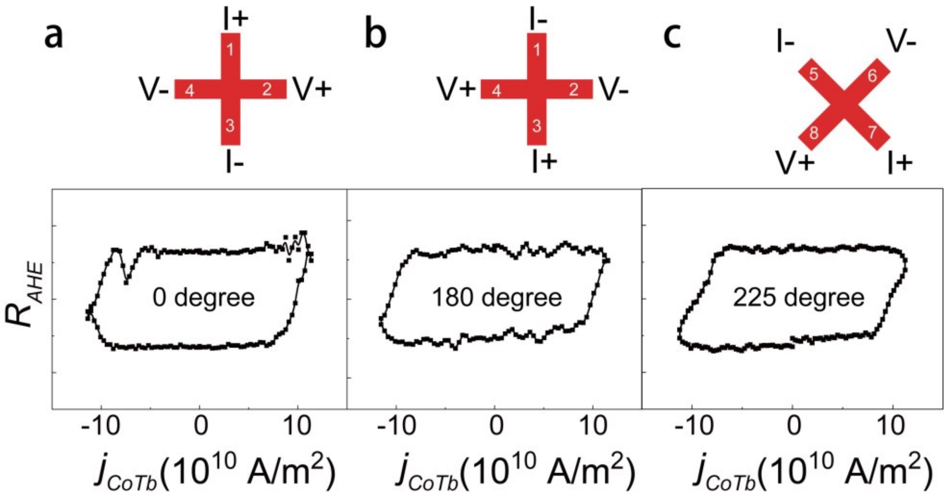

Fig. S13 SOT switching loops of previously untrained devices at different angles: **a,** 0 degree. **b,** 180 degree. **c,** 225 degree.

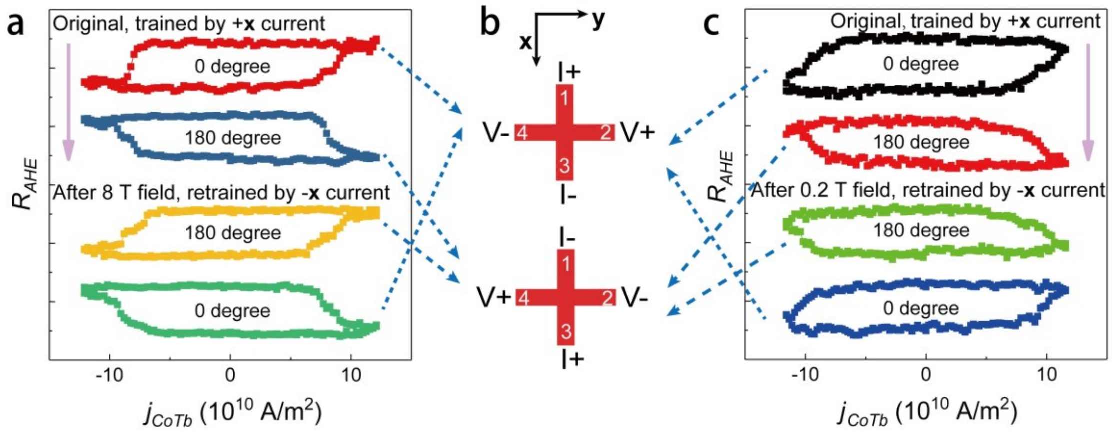

Fig. S14 Device Retraining. a, Current-induced switching loops before and after re-initializing the device with an 8 T magnetic field and retraining with a current in the –x direction. **b,** Schematic illustration of 0 degree and 180 degree measurement configurations. **c,** Current-induced switching loops before and after attempted re-initialization of the device with a 0.2 T magnetic field, which is not large enough to allow for retraining with a current in the –x direction.

S10. Device uniformity across a 100 mm wafer

We performed additional experiments to investigate the device-to-device uniformity not only within a single chip, but also across a wafer. To do so, we performed field-free switching experiments in devices randomly picked on a full 100 mm (4 inch) silicon wafer. The results are shown in Fig. S15, where parts 2, 4, 6 and 8 on the wafer (indicated in Fig. S15a) were selected for measurements. Fig. S15b plots the field-free current-induced switching loops of the devices on these different chips using the same measurement setup. Clearly, all measurements show the same switching polarity as well as similar critical switching currents. This device-to-device uniformity over a large area further reveals that very little in-plane concentration variations can exist in our samples. These results are also encouraging for the translation of this field-free SOT switching scheme to industrial manufacturing on larger wafer sizes.

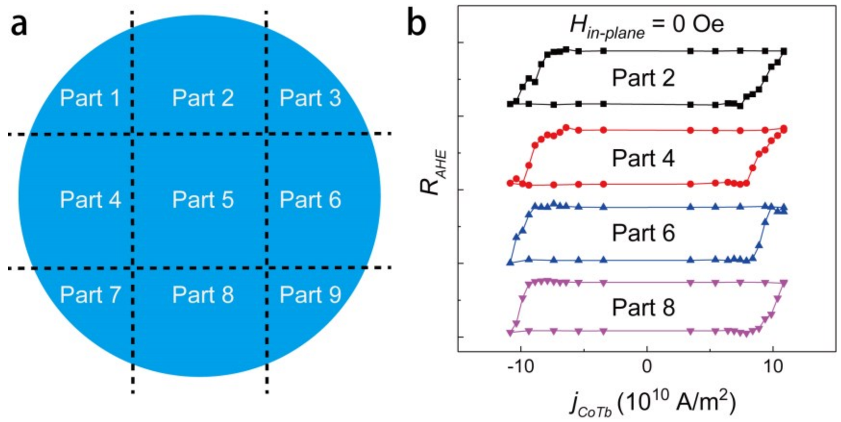

Fig. S15 Field free switching experiments on a full 100 mm wafer. a, Die layout and numbering of a 100 mm (4 inch) wafer. **b,** Current-induced SOT switching loops of devices in parts 2, 4, 6 and 8 on the wafer under zero in-plane field. All the devices on the wafer show the same switching behavior, indicating good device-to-device uniformity across the wafer.

References

[1] Lee K. S., Lee S. W., Min B. C. & Lee K. J. Threshold current for switching of a perpendicular magnetic layer induced by spin Hall effect. *Appl. Phys. Lett.* **102**, 112410 (2013).

[2] Hals, K. M., Tserkovnyak, Y. & Brataas, A. Phenomenology of current-induced dynamics in antiferromagnets. *Phys. Rev. Lett.* **106**, 107206 (2011).

[3] Manchon, A. et al. Current-induced spin-orbit torques in ferromagnetic and antiferromagnetic systems. *Rev. Mod. Phys.* **91**, 035004 (2019).

[4] Sánchez-Tejerina, L. et al. Dynamics of domain-wall motion driven by spin-orbit torque in antiferromagnets. *Phys. Rev. B* **101**, 014433 (2020).

[5] Martínez, E., Raposo, V. & Alejos, Ó. Current-driven domain wall dynamics in ferrimagnets: Micromagnetic approach and collective coordinate's model. *J. Magn. Magn. Mater.* **491**, 165545 (2019).

[6] Sánchez-Tejerina, L. et al. Unified Framework for Micromagnetic Modeling of Ferro-, Ferri-, and Antiferromagnetic Materials at Mesoscopic Scale: Domain Wall Dynamics as a Case Study. *IEEE Magn. Lett.* **11**, 1-5 (2020).

[7] Liu, L., Moriyama, T., Ralph, D. C. & Buhrman, R. A. Spin-torque ferromagnetic resonance induced by the spin Hall effect. *Phys. Rev. Lett.* **106**, 036601 (2011).

[8] Heide, M., Bihlmayer, G. & Blügel, S. Dzyaloshinskii-Moriya interaction accounting for the orientation of magnetic domains in ultrathin films: Fe/W (110). *Phys. Rev. B* **78**, 140403 (2008).

[9] Martinez, E., Emori, S. & Beach, G. S. Current-driven domain wall motion along high perpendicular anisotropy multilayers: The role of the Rashba field, the spin Hall effect, and the Dzyaloshinskii-Moriya interaction. *Appl. Phys. Lett.* **103**, 072406 (2013).

[10] Tomasello, R. et al. Domain periodicity in an easy-plane antiferromagnet with Dzyaloshinskii-Moriya interaction. Preprint at: http://arXiv.org/cond-mat/2004.01944 (2020).

[11] Siddiqui, S. et al. Current-induced domain wall motion in a compensated ferrimagnet. *Phys. Rev. Lett*. **121**, 057701 (2018).

[12] Finley, J. & Liu, L. Spin-orbit-torque efficiency in compensated ferrimagnetic cobalt-terbium alloys. *Phys.Rev. Appl.* **6**, 054001 (2016).

[13] Wang, H. et al. Spin-orbit-torque switching mediated by an antiferromagnetic insulator. *Phys. Rev. Appl*. **11**, 044070 (2019).

[14] Je, S. G. et al. Spin-orbit torque-induced switching in ferrimagnetic alloys: Experiments and modeling. *Appl. Phys. Lett.* **112**, 062401 (2018).

[15] Zhang, R. Q. et al. Current-induced magnetization switching in a CoTb amorphous single layer. *Phys. Rev. B* **101**, 214418 (2020).

[16] Yu G. et al. Switching of perpendicular magnetization by spin–orbit torques in the absence of external magnetic fields. *Nat. Nanotechnol*. **9**, 548 (2014).

[17] Chen, T. Y., Chan, H. I., Liao, W. B. & Pai, C. F. Current induced spin-orbit torque and field-free switching in Mo-based magnetic heterostructures. *Phys. Rev. Appl.* **10**, 044038 (2018).

[18] van den Brink, A. et al. Field-free magnetization reversal by spin-Hall effect and exchange bias. *Nat. Commun.* **7**, 1-6 (2016).

[19] Cai, K. et al. Electric field control of deterministic current-induced magnetization switching in a hybrid ferromagnetic/ferroelectric structure. *Nat. Mater.* **16**, 712-716 (2017).

[20] Sun, J. Z. et al. Spin-torque switching efficiency in CoFeB-MgO based tunnel junctions. *Phys. Rev. B* **88**, 104426 (2013).

[21] Liu, Y. T. et al. Determination of Spin-Orbit-Torque Efficiencies in Heterostructures with In-Plane Magnetic Anisotropy. *Phys. Rev. Appl*. **13**, 044032 (2020).

[22] Hu, C. Y. & Pai, C. F. Benchmarking of Spin–Orbit Torque Switching Efficiency in Pt Alloys. *Adv. Quantum Technol*. 2000024 (2020).

[23] Chen, B., Lourembam, J., Goolaup, S. & Lim, S. T. Field-free spin-orbit torque switching of a perpendicular ferromagnet with Dzyaloshinskii-Moriya interaction. *Appl. Phys. Lett*. **114**, 022401 (2019).

[24] Wu, K., Su, D., Saha, R. & Wang, J. P. Deterministic field-free switching of a perpendicularly magnetized ferromagnetic layer via the joint effects of the Dzyaloshinskii–Moriya interaction and damping-and field-like spin–orbit torques: an appraisal. *J. Phys. D Appl. Phys.* **53**, 205002 (2020).

[25] Dai, M. & Hu, J. M. Field-free spin–orbit torque perpendicular magnetization switching in ultrathin nanostructures. *NPJ Comput. Mater.* **6**, 1-10 (2020).

[26] Lee, J. M., et al. Field-free spin–orbit torque switching from geometrical domain-wall pinning. *Nano Lett.* **18**, 4669-4674 (2018).

[27] Fert, A. & Hippert, F. Anisotropy of spin-glasses from torque measurements. *Phys. Rev. Lett.* **49**, 1508 (1982).

[28] Fert, A., Arvanitis, D. & Hippert, F. Triad anisotropy of spin glasses and torque experiments. *J. Appl. Phys.* **55**, 1640-1645 (1984).

[29] Fert, A. & Levy, P. M. Role of anisotropic exchange interactions in determining the properties of spin-glasses. *Phys. Rev. Lett.* **44**, 1538 (1980).

[30] Levy, P. M. & Fert, A. Anisotropy induced by nonmagnetic impurities in CuMn spin-glass alloys. *Phys. Rev. B* **23**, 4667 (1981).

[31] Hippert, F., Alloul, H. & Fert, A. Anisotropy energy of CuMn spin glasses. *J. Appl. Phys.* **53**, 7702-7704 (1982).
